# Medical Humanities Education and Its Influence on Students' Outcomes in Taiwan: A Systematic Review

**DOI:** 10.3389/fmed.2022.857488

**Published:** 2022-05-16

**Authors:** Bao Lan Hoang, Lynn Valerie Monrouxe, Kuo-Su Chen, Shu-Ching Chang, Neville Chiavaroli, Yosika Septi Mauludina, Chien-Da Huang

**Affiliations:** ^1^Chang Gung Medical Education Research Centre (CG-MERC), Chang Gung Memorial Hospital, Linkou, Taiwan; ^2^Faculty of Medicine and Health, Susan Wakil Health Building, The University of Sydney, Sydney, NSW, Australia; ^3^Department of Nephrology, Chang Gung Memorial Hospital, Keelung, Taiwan; ^4^Department of Medical Humanities and Social Sciences, Chang Gung University College of Medicine, Taoyuan, Taiwan; ^5^Department of Pathology, Chang Gung Memorial Hospital, Linkou, Taiwan; ^6^Australian Council for Educational Research, Melbourne, VIC, Australia; ^7^Department of Medical Education and Thoracic Medicine, Chang Gung Memorial Hospital, Chang Gung University, Taipei, Taiwan

**Keywords:** medical humanities, medical students, nursing students, medical education, systematic review, curriculum

## Abstract

**Background:**

Medical education has emphasized the importance of integrating medical humanities training into the curriculum to benefit medical and nursing students' future practice, featuring in the list of national funding priorities for healthcare education research in Taiwan for many years. However, the extent to which this drive has resulted in medical humanities training, what rationales underpin its inclusion, and its efficacy is largely unknown. This study aims to address these issues across medical humanities programs within the Taiwanese context.

**Methods:**

We conducted a systematic review. Inclusion criteria included studies in English or Mandarin reporting outcomes of medical humanities courses in healthcare education settings in Taiwan between 2000 and 2019. We searched across five electronic databases (PubMed, Embase, ERIC, PsycInfo, Web of Science), following PRISMA guidelines. The Best Evidence Medical Education (BEME) Global Scale and Kirkpatrick Levels are used for identifying the strength of evidence.

**Results:**

17 articles were extracted from the 134 identified. Intrinsic and instrumental rationales for the inclusion of medical humanities education were common, compared with epistemological-based and critical-based approaches. Several positive impacts were identified in relation to participation including modification of attitudes, knowledge, and skills. However, the highest level (i.e., unequivocal) of evidence characterized by effects on students' behaviors or ongoing interaction with colleagues and patients is lacking.

**Conclusion:**

Findings suggest that although medical humanities education is widely implemented in Taiwan, no clear consensus has been reached regarding the rationale for inclusion or how it is localized from Western to Asian contexts. Future research still needs to explore the long-term impact of medical humanities education for medical and nursing students and its impact on patient care.

**Systematic Review Registration:**

https://www.crd.york.ac.uk/PROSPERO, identifier: CRD42019123967.

## Introduction

While advances in scientific and technical knowledge have contributed to considerable progress in medicine and health, it has been argued that clinical practice remains as much an art as science (1–3). This perspective has contributed to the development of the field known as the *medical humanities* which support and inspires the application of the humanities for teaching medical and nursing students. The inclusion of the medical humanities into medical curricula has to date been driven by four key rationales: *instrumental, intrinsic, critical*, and *epistemological* (3). Despite this range of rationales for its inclusion, there remains a lack of consensus regarding the impact and value of the medical humanities in terms of fulfilling its expected roles in medical curricula. Furthermore, reviews of the medical humanities have assumed a predominately western perspective, ignoring how the medica humanities are developed and implemented more globally (4–13). The aim of this systematic review is therefore to partially fill this gap in the literature by ascertaining the different rationales for delivering medical humanities programs in an Asian context, specifically Taiwan, and the extent to which it is effective to those ends.

### What Is Medical Humanities?

Although there is no consensus for the definition of the medical humanities, it commonly includes an interdisciplinary perspective that draws on both creative and intellectual methodological aspects of disciplines such as anthropology, art, bioethics, drama and film, history, literature, music, philosophy, psychology, and sociology (14, 15). And while there have been multiple attempts to define what is meant by the term *medical humanities* (9, 15–19), conceptualizations tend to cluster under four key rationales: *instrumental, intrinsic, critical*, and *epistemological*. Thus, the intrinsic (or non-instrumental) rationale focuses on the potential counterbalancing effect of bringing a humanistic perspective into the curriculum (17), whereas the instrumental (or practical) rationale emphasizes the knowledge, skills, and attitudes that are directly related to clinical practice (e.g., communication, empathy, narrative competence, etc.) (3, 17). The critical rationale utilizes the humanities to bring an analytical and questioning lens to education and health practices (7, 20, 21). Finally, the epistemological rationale aims to explain how the humanities disciplines, and their methods of inquiry, are fundamental to medical pedagogy and practice (22, 23).

### Efficacy of the Medical Humanities

In an era of outcome-based education, and to justify the expense of its inclusion, it is important for the medical humanities community to address the need for empirical evidence of its effectiveness (7). Research suggests that benefits for the inclusion of the medical humanities in undergraduate medical curricula comprise enhanced empathy, cultural awareness, observational skills, teamwork, reasoning, listening, self-reflection, communication skills, and reduced stress (24–26). However, evidence for any positive long-term impact for medical students themselves, and ultimately for patient care, is sparse (7).

### About the Systematic Reviews

This study, to our knowledge, is the first systematic review to focus on the relative effectiveness of nationwide medical humanities programs. It is also unique in its inclusion of both Taiwanese undergraduate and postgraduate medical and nursing curricula, as well as in its attempt to ascertain the extent to which the previously identified rationales for the medical humanities are present in this context. In doing so we ask the following research questions (RQs):

RQ1: How are the medical humanities defined in Taiwan and what rationales are used for their inclusion in medical and nursing curricula?
RQ2: What types of medical humanities interventions are employed in the Taiwan medical and nursing curricula?
RQ3: How are the medical humanities outcomes assessed across Taiwan's medical and nursing curricula?
RQ4: On what type of evidence is the successful delivery of the medical humanities in Taiwan based?
RQ5: To what extent are medical humanities curricula successful in delivering specific outcomes?

## Methods

We conducted a systematic review focusing on medical humanities education interventions in medical and nursing education in Taiwan. We used the Best Evidence Medical Education (BEME) Global Rating Scale and Kirkpatrick-based outcomes (Online Appendix 2) to evaluate the strength of the evidence.

BEME is defined as: “the implementation by teachers and educational bodies in their practice, of methods and approaches to education based on the best evidence available.” BEME can be considered as a spectrum ranging from 100% opinion-based education where there is no useful evidence, to 100% evidence-based education where there is adequate evidence (27).

The Kirkpatrick (1996) model (28), additionally, can provide techniques for appraisal of the evidence for any reported training program and could be used to evaluate whether such training program is likely to meet the needs of requirements of both organizers (teachers, university, hospital) and participants (students'). There are 4 levels in this model to evaluate training comprising *reaction (1), learning (2), behavior (3)*, and *results (4)*. The first level of evaluation, *reaction*, typically involves trainees completing a post-course evaluation of their impressions of the program. Such evaluation does not measure what participants have learned, but gauges the interest, motivation, and attention levels of participants. The second level, *learning*, involves measuring what participants have learned in terms of both knowledge and/or skills. Learning evaluation can include trainees participating in written assessments or role-plays to demonstrate their skills. This level of evaluation allows participants to demonstrate their understanding of specific skills and/or knowledge within the learning program. The third level is *behavior* or performance. This involves assessment of the trainee's ability to use their newly learned knowledge or skills in the workplace. This level of evaluation attempts to determine whether participants (who may already have demonstrated acquisition of specific skills and/or knowledge) use their new skills when they return to the work environment. The fourth level, described as *results*, is a measure of the impact that the training has had overall, including financial or morale impacts. This might include improvement in, for example, staff–resident interaction, decreased incidents of challenging behavior, and staff turnover (28).

### Context

This study focused on the implementation of medical humanities into undergraduate medical and nursing education curricula in Taiwan (note, in nursing education the term “medical humanities” is also used). Specifically, in Taiwan, most medical and nursing schools adopt definitions of the medical humanities that have been developed in Anglo-American contexts, such as the mission statement developed by New York University (NYU), which defines medical humanities as “an interdisciplinary field of humanities (literature, philosophy, ethics, history, and religion), social science (anthropology, cultural studies, psychology, sociology), and the arts (literature, theater, film, multimedia, and visual arts) and their application to healthcare education and practice” (18, 29). Following reviews by the United States National Committee on Foreign Medical Education and Accreditation in 1998, Taiwan's medical schools initiated curricular reform in 2002. Specifically, they prescribed humanities education for entering medical and nursing students compared to that required in the United States (30, 31).

### Search Strategy

Our systematic review was executed in two phases: first, we searched electronic databases; second, the authors then manually searched reference lists for relevant articles. Articles in the first phase were obtained from the following electronic databases: PubMed, Embase, ERIC, PsycInfo, and Web of Science (See Appendix 1). We limited our search to articles published from 2000 onwards, to align with the development of the medical humanities in Taiwan (30, 31). Once we reached a consensus about the search terms, one author (HBL) ran an initial search (December 1, 2018), which was repeated a second time (May 31, 2019). After removing duplicates, 134 articles remained. Following this, we examined the reference lists of these articles for further relevant sources. We also examined the works cited in previous systematic reviews on medical humanities education in Taiwan to identify any additional articles that could be relevant to our research questions and within the range of our study criteria.

### Article Selection

**Figure 1:**
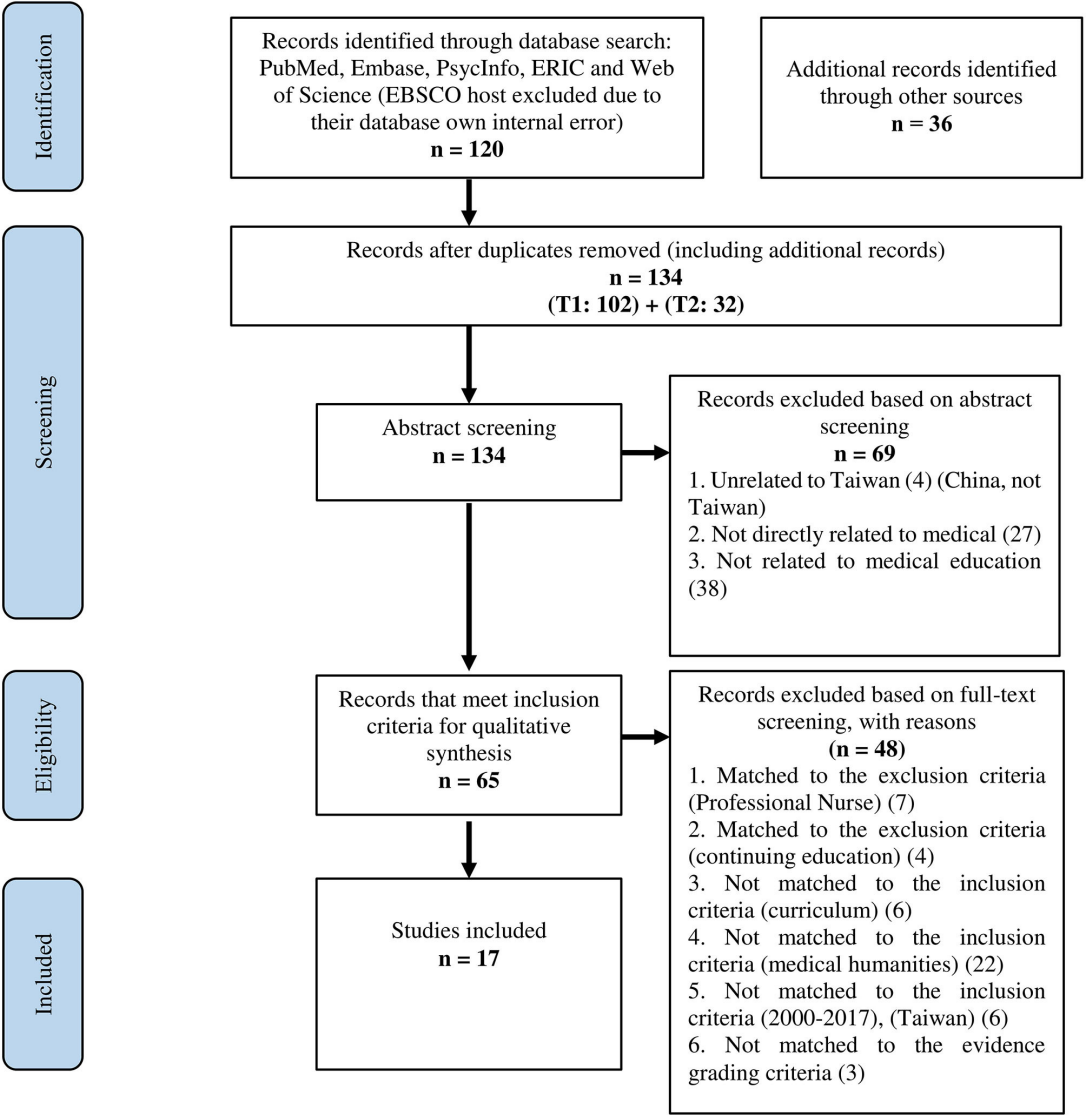
PRISMA flow diagram of literature search.

All researchers independently identified relevant articles for full-text review in Endnote by scanning the titles and abstracts on the basis of the inclusion and exclusion criteria set out in Box 1. As this systematic review focused on the undergraduate curricula, research conducted with continuing students [trainees], post-graduate students [trainees], and professional nurses [medical practitioners] and non-degree courses/further professional training (continuing education) were excluded. We limited the lower-range of publication to the year 2000 as medical education is a relatively new field in Taiwan, with focussed funding beginning in 2007 (32). Furthermore, during the past 20 years the Medical Humanities have become a focus of this funding, resulting in a rise in related publications in 2016 (32). We limit the languages to English and Mandarin for two reasons. First, these are the languages that Taiwanese education researchers in the medical humanities and medical education fields mainly use for publication. Second, while it might be possible that researchers use other languages (i.e., French), it is impossible for our team to read them as we have no expertise in this. Finally, there studies are geographically limited to Taiwan. Studies of other geographic locations will be excluded. BLH conducted a full-text analysis for eligibility. Seventeen studies reporting on medical humanities education in Taiwan were included in the final analysis. Figure 1 contains a PRISMA flow diagram of the search and selection process.

Box 1 **Inclusion and exclusion criteria.**

**Inclusion criteria**

- Date range: 1^st^ January 2000 - 31^st^ May 2019
- Population: Medical Student [Clerk, Intern], Medical Teacher [Trainer, Educator], Nursing Student, Nursing Course, Medical School [College, University], Medical Course
- Exposure: Medical Humanities, Narrative Medicine, Health Humanities,
- Outcome: Participants' inter-professional collaboration skills, patient-centered decision on professional issues, Participants' cognition on medical humanities, Participants' improvement of doctor-patient communication, cultural competence, critical thinking and in-field clinical performance after medical humanities training
- Language: English, Mandarin
- Geographic location: Taiwan
- Setting: Medical School [College, University], Nursing School, Hospital
- Study design: All studies with empirical data

**Exclusion criteria**

- Date range: Before 1^st^ January 2000
- Population: Continuing Student, Continuing Trainee, Professional Nurse
- Exposure: Continuing Education, Post-graduate Student
- Language: Other than English and Mandarin
- Geographic location: Other than Taiwan
- Study design: Systematic reviews or reviews

### Data Extraction Process

Data extraction comprised the following process: We managed the coding of articles in ATLAS.ti (version 8.0) software. After all seventeen studies were imported to the library, BLH screened them once again to ensure all inclusion and exclusion criteria had been applied correctly. Following this, the coding began: noting firstly the author(s) name(s), year of publication, and language. A second-team member (CDH) double-checked the database. BLH then coded for study design, research period, stages of the training, type of participants, research specific outcomes, and other specific information required to answer the research questions. Another team member (LVM) then checked the ATLAS.ti database as an independent review, in addition to whole-team discussions on process during our regular meetings online and on-site. Discussions regarding the rationales used for medical humanities inclusion in the curricula taken within each article occurred online, via emails. Discrepancies were communicated and resolved. Evidence grading was undertaken by BLH who categorized articles according to the Best Evidence Medical Education (BEME) Global Rating Scale and Kirkpatrick-based outcomes (Online Appendix 2). The same researcher then sent this analysis to all co-authors for independent verification. Once this step was completed, all co-authors discussed and compared their scores, determined agreement, and resolved any disagreements.

To answer the key research questions, data were deductively coded according to the following criteria: (1) presence and origin of definitions for medical humanities; (2) rationales for implementation of the medical humanities (outlined earlier).

## Results

We present the main body of our results section according to the research questions. In terms of study participants, we note that fourteen articles comprised only medical students as participants and two articles had both medical and nursing students (33, 34) creating an interdisciplinary team. Only one study (35) included a diversified and interdisciplinary team of participants including medical, nursing, economics, chemistry, mechanical engineering and architecture, mathematics, life science and informatics students'. It should also be noted that there were no studies with patients as participants. Specific details of each paper are included in Tables 1, 2.

**Table 1:** Extraction of medical humanities sources of definitions, types of interventions, intentions and the relevance of the researches to Taiwan's medical humanities education.

**Reference**	**Medical humanities definition source**	**Rationale (s)**	**Type of intervention**	**Intention**	**Relevance to humanities**
Yang et al. (26)	USA	Instrumental	Exposure to visual art	To increase empathy, cultural awareness, observational skills, better team-work, communication skills and stress reducing.	Students' will have to understanding arts through persons and context within it
Tsai (40)	USA	Instrumental	Expose to elderly community care practice	To increase social trust and change the relationship with patients	Incorporating the concept of “doctor as mediator in the changing relationship with patients”
Yang and Yang (24)	USA & Germany	Instrumental	Exposure to visual art	To increase empathy and sensitivity	Students' will have to understanding arts through persons and context within it
Wong et al. (42)	USA & EU	Intrinsic	Field work after informal and formal humanities training	To see the importance of several ways of learning medical humanities informally.	Field work with its interaction with real patients made the course more authentic to the students'
Wang et al. (45)	Not specified	Instrumental	Narrative/Storytelling	To enhance medical care students sense of meaning in life and critical thinking capacity	Cultivating professional and humanistic attitude
Tseng and Lin (36)	USA	Intrinsic	Course	To make a change in the way of thinking/participants' emotion	Cultivating professional and humanistic attitude
Lin et al. (43)	USA and Canada	Instrumental	Reflective writing practice and receiving feedback from mentor	To increase participants' clinical observation skills, empathetic listening skills, interpersonal and communication skills, and problem-solving abilities	Letting students' have a diversified, flexible thinking understand patients' perspective better
Kan et al. (35)	USA and UK	Intrinsic	Course	To explore the importance of life perspectives such as philosophies of life, which should help them treat end-stage patients with more humanistic passion	Students' are asked to think about life and death from humanistic viewpoint
Huang et al. (37)	USA	Instrumental	Narrative/Storytelling	To increase empathy and be more human-focused	The importance of “medicine as an art for human healing” is raised.
Fan et al. (38)	USA	Epistemological	Course	Integrated course in psychiatry and literature to increase medical students' grades in the later psychiatry courses	Training medical students' to think in a humanistic way, compared to the traditional clinicians' ways.
Yang et al. (33)	Australia	Instrumental	Exposure to elderly community care practice	To increase empathy, communication and collaboration skills	Responding to patient emotions and strengthening the patient-physician relationship to increase the social trust
Liao and Wang (25)	Not specified	Instrumental	Reflective writing practice and receiving feedback from mentor	To enhance students' empathy, facilitate interdisciplinarity and connect patients' diseases to social/cultural contexts	Literature as a vehicle for exploring what it means to be humane
Cheng et al. (44)	USA and World Federation for Medical Education	Intrinsic	Course	To make participants value the profession more	Solving the lack of commitment to the profession of several professionals currently due to bad relationship with patients
Chen and Chou (39)	USA	Instrumental	Field practice program	To improve medical intellectual and communication skills and also for developing humanitarian nature in medical professionals.	Educating the history of medicine with authentic stories so that students' will be more cognitively human-focused.
Chiou et al. (41)	UK, Romania, Spain and Hungary	Intrinsic	“Silent mentor” (death human body) initiation ceremony	To see higher humanistic consideration in participants	Strengthening student's medical humanity and learning attitudes
Tsai et al. (46)	Not specified	Intrinsic	Course	To increase ethical decision making	The humanities perspective of palliative care
Lin et al. (34)	WHO	Critical	Course with problem-based learning, lectures and feedback	To increase students' inter-collaboration and problem-solving skills	Creating a better inter-collaboration between future nursing and medical professionals so that they will agree on the ethical decision making and values

**Table 2:** Extraction of constructs and assessments in Taiwan medical curricula, research methodologies used and participants of Taiwan's medical humanities articles.

**Reference**	**Construct**	**Assessment**	**Participant**	**Methodology**
Yang et al. (26)	Participants' understanding of people and arts in contexts	Participants' written records and clinical teachers' direct observation and notes on students' discussions	Medical students	Qualitative, observation and the written feedback from students'
Tsai (40)	Students' community in-field practice and the community development	Participants' self-assessment of achievements in communication skills	Medical students	Qualitative, observation, quasi-longitudinal
Yang and Yang (24)	Participants' understanding of people and arts in contexts	Participants' empathy development after the course via faculty observation and their discussions	Medical students	Quantitative, questionnaire, pre-tests and post-tests
Wong et al. (42)	Students' performance during internship after informal learning model	Students' behavior, observations of senior colleagues and educators, and their intentions of learning	Medical students	Qualitative, field notes and interview analysis
Wang et al. (45)	Participants' critical thinking competence and awareness on sense of life	Triad of attention, representation, and affiliation in their close reading and reflective writing, along with a summary or description.	Medical students	Quantitative, questionnaire, pre-tests and post-tests
Tseng and Lin (36)	Students' experiences and attitudes about death	Students' responses to interview questions about the experience and their coping strategies	Medical students	Qualitative, Semi-structured, focus group interviews, Observation
Lin et al. (43)	Participants' word usage	Reflective narratives	Medical students	Quantitative, questionnaire, pre-tests and post-tests
Kan et al. (35)	Participants' emotions toward the death	Unscheduled short tests and reports on field trips	Medical students, nursing students, non-medical/nursing related students'	Qualitative, report and written assignment
Huang et al. (37)	Students' perceptions about the narrative medicine activity and its progress model	Clinical stories in their narrative writing assignments in different ways, such as story-telling or poetry-reading	Medical students	Quantitative, cross-sectional questionnaire, pre-tests and post-tests
Fan et al. (38)	Socioeconomic status, mental health and physical health, academic performances	Students' mental and physical health, academic grades and faculty observation	Medical students	Quantitative, quasi-longitudinal, baseline survey, students' academic performance scores
Yang et al. (33)	Participant's listening and communication skills	Meeting to share opinions/feelings on the services offered, final reports on achievements and difficulties, solutions to problems, progress made, issues and ways to improve the course	Medical students and nursing students	Qualitative, interview analysis, observation
Liao and Wang (25)	Students' empathy, critical thinking, and reflective writing	Reflection per week, discussion forum and presentation	Medical students	Quantitative, questionnaire, pre-tests and post-tests
Cheng et al. (44)	Students' knowledge regarding medical ethics and laws, and doctor-patient communication	Students' improvement of knowledge on medical ethics and laws, and doctor-patient communication	Medical students	Mixed quantitative and qualitative, questionnaire, pre- and post-test and written feedback collection
Chen and Chou (39)	Communication competence and humanitarian nature	Participants' cognition of medical history and guiding presentation	Medical students	Quantitative, questionnaire, pre-tests and post-tests
Chiou et al. (41)	Participants' emotions toward the death	Responses to questions love and care of participants toward patients	Medical students	Quantitative, questionnaire, pre-tests and post-tests
Tsai et al. (46)	Students' knowledge of palliative care and their beliefs concerning ethical decision-making in palliative care	Responses to questions about knowledge of palliative care and to questions about ethical decision-making in palliative care	Medical students	Quantitative, cross-sectional survey, pre-tests and post-tests
Lin et al. (34)	Students' attitude toward interprofessional collaboration	Students' self-assessments on their confidence and attitude toward interprofessional collaboration after the course, and multi-perspective written texts on professional issues	Medical students and nursing students	Quantitative, cross-sectional survey, pre-tests and post-tests

### RQ1: How Is the Medical Humanities Defined in Taiwan and What Rationales Are Used for Their Inclusion in Medical Curricula?

Most of the studies applied Western definitions of the medical humanities. In particular, six (*n* = 6; 35.30%) (26, 36–40) used solely the New York University's medical humanities definition. Eight studies (*n* = 8; 47,05%) (24, 33–35, 41–44) applied other Western definitions from one or more of the following countries: USA, UK, Spain, Romania, Australia, or organizations such as World Federation for Medical Education, WHO and EU. A minority of studies (*n* = 3; 17.65%) did not clearly specify the definition they used (25, 45, 46).

In terms of rationales for applying the medical humanities to medical and nursing education, we identified studies that could be classified into the four rationales (as outlined earlier) (3), albeit to varying degrees. In terms of specific emphases (though noting some overlap), nine articles highlighted evidence relating to the instrumental rationale (24–26, 33, 37, 39, 40, 43, 45). Articles classified as drawing on the instrumental rationale frequently stated explicitly that their pedagogical aims were to use arts as a tool to develop students' competencies as physicians, such as increased empathy and cultural sensitivity (24–26, 43), enhancing students' listening and communication skills (26), and facilitating cooperation with students' in other departments (25, 33). However, other articles classified under this rationale highlighted the importance of specific effects that were felt or gained by students' through the program, such as increasing their task responsibility (37, 39, 40, 43, 45), social interaction and trust (40, 43), and self-development and reflection (37, 45).

Six articles were classified according to an intrinsic rationale (35, 36, 41, 42, 44, 46). Most of these articles explicitly introduced the humanities perspective according to (1) the three principles of primacy of patients' welfare, autonomy and social justice (42); (2) the relationship between “detachment” and “concern” (36), facilitating an understanding about the meaning of sickness and death in life (35), and learning through a “silent mentor” (body donation) to develop positive attitudes toward death (41). While three other articles emphasize the potential counterbalancing effects of the humanities in the medical curriculum (42, 44, 46), thus aiming to facilitate a more patient-focused student (42) and greater understanding of the ethical dimension of clinical practice (44, 46).

Only one article was classified to the critical rationale (34): valuing and applying the humanities' methods of interdisciplinarity, rather than simply drawing on narrative texts as sources of patient or practitioner perspectives. Here, differences among groups toward interprofessional communication and collaboration were drawn out (34). For example, drawing on evidence from interview participants the authors argue that an “interprofessional PBL curriculum would be a good and feasible approach for students' to foster communication and collaboration skills for solving inter-professional conflicts of value” (p.506).

The epistemological rationale was also represented by a single paper (38). In this paper, the humanities are used to represent characteristic ways of understanding and reasoning which are highly relevant to medical practice, with a focus on the particular, tolerance of ambiguity, and access to others' perspectives. To illustrate, the authors argue that “literature forces us to think in a way that we in the medical field may not be accustomed to… opens new doors, new worlds, worlds of metaphors and hyperboles, similes and symbolism. … [and] creates a personal connection between the reader and the characters” (p.477).

### RQ2: What Types of Medical Humanities Interventions Are Employed in the Taiwan Medical Curricula?

For articles based on the instrumental rationale, exposure to visual arts (24, 26), narrative/storytelling (37, 45), reflective writing practice and feedback (43), exposure to elderly community care practice (33, 40), fieldwork (39) and a course (44) were employed as interventions. Exposure to elderly community care practice or fieldwork is used by Tsai (40), Yang et al. (33) and Chen and Chou (39). The aim here is the promotion of empathy and communication, as well as inter-collaboration skills of medical/nursing students.

Within the intrinsic articles, fieldwork (42), memorial ceremony (41) and a course (35, 36, 44, 46) were used as interventions to increase awareness primarily related to ethical issues and participants' own human nature. Specifically, courses here can vary from a series of sessions to a single workshop. A course intervention was also used in the critical article (34). Here, a series of sessions were employed to intervene, discuss and help students' solve ethical professional dilemmas. The epistemological-based article (38) reported an intervention that comprised an integrated course in psychiatry and literature that was used to show how the humanities disciplines, and their methods of inquiry, are fundamental to medical pedagogy and how it can increase students' performance in medical professional skills training.

### RQ3: How Are Medical Humanities Outcomes Assessed Across Taiwan's Medical Curricula?

The outcomes of medical humanities interventions are assessed via a range of methods. Self-assessments about participants' medical humanities skills development/professional development (5), faculty observation (47), and scheduled and unscheduled tests at different points within the study (35, 38). Specifically, five articles (26, 33, 37, 43, 45) assessed learning via written assignments including self-reflection and feedback (26), reflective writing (33, 43), narrative writing (37, 45).

As for the evaluation of participants' medical humanities skills development/professional development, while self-assessment of students' own perceptions on their development was used in two articles (34, 40), two papers reported assessing medical humanities constructs via quantitative questionnaire responses objectively (39, 46). Furthermore, participants' narratives were used to assess the appreciation of the medical humanities (36, 41, 43, 44). In addition, faculty observations of student's discussions (for assessment), empathy, professional behavior, intentions for learning or cognitive skills, and mental or psychological health of students, was also used (24, 26, 38, 40, 42). Finally, two articles assessed medical humanities constructs via scheduled and unscheduled tests at different points within the study (35, 38).

### RQ4: On What Type of Evidence Is the Successful Delivery of the Medical Humanities in Taiwan Based?

We considered the type of research study that was undertaken. In terms of methodology, two studies used mixed methods with pre-test and post-test outcomes and collection of written feedback (26, 44). Five articles (33, 35, 36, 40, 42) applied a variety of qualitative data collection methods. For example, participant observation (33, 40), field notes (42), semi-structured focus group interviews (36), and one-to-one interviewing (33). Ten articles drew on quantitative methods comprising pre- and post-test questionnaires (24, 25, 34, 37, 39, 41, 43, 45, 46) and a combination of a baseline survey and students' academic performance scores (38). Additionally, two articles utilized quasi-longitudinal studies (38, 40). Tsai (40) conducted a curriculum assessment focusing on essential background knowledge and methodology during 2 years (Stage 1), and a program akin to community health building camp volunteer training (Stage 2). Fan et al. (38) used a quantitative, quasi-longitudinal, cross-sectional study over 3 years. At the time of entrance to medical school (first year), these students completed a thorough baseline survey with questions related to their socioeconomic status, mental health and physical health. Students' academic performance including medical school grade point averages (GPAs), merits, demerits, medical school admissions interview scores, and scores on the national entrance examination were also collected. Merit and demerit points were a supplementary evaluation system, provided by faculty for positive or negative student behaviors respectively. Students then had the option of taking the “Psychiatry and Western Literature” course during their first year of medical school, resulting in two groups of students (those taking, and those not). Following completion of the fourth year, researchers examined the baseline data for statistically significant differences between the two groups.

### RQ5: To What Extent Are Medical Humanities Curricula Successful in Delivering Specific Outcomes?

In this section we report on the extent to which the included studies met the quality criteria using the BEME Strength of Evidence Scale (48), rating them as either level 1, 2, 3, or 4 accordingly. In doing so we took into account a number of factors, such as the **Quality** of the research evidence available, the **Utility** of the evidence, the **Extent** of the evidence, the **Strength** of the evidence, the **Target** or outcomes measured, and the **Setting** or context. Table 3 provides an overview of our strength of the evidence classifications, noting that some studies cut across different levels due to multiple measures. We structure the main body of this section by commenting on the level at which they measured outcomes (according to Kirkpatrick's criteria) and the relative success of these outcomes. Again, articles cut across these levels according to the measurements commented upon.

**Table 3:** BEME strength of the evidence summary.

**BEME strength of evidence level**	**Articles**
1: An absence of any clear and significant changes	Three studies: research used the JSPE to measure empathy (24), the CLIWC (Chinese version of Linguistic Inquiry and Word Count) to measure the psychological process on reflective writing (43), and course assessment (35). All failed to find any significant differences as a result of the study.
2: Weak/ambiguous results, although trends identified	Four studies: results suggest that participants are aware of the medical humanities, developed some new skills and/or changed their attitudes toward the importance of medical humanities. However, no specific action or significant evidence of application to real clinical settings is identified (34, 36, 39, 46).
3: Results are sufficiently robust to form a basis for conclusions.	Five studies: in particular, these articles suggest that students took action to improve their treatment quality toward patients, applying a humanistic approach toward them (33, 38, 40, 42, 44). Data also suggests that students' behaviors and thoughts change (as specified in level 2). However, research designs did not include pre-/-post-test, or measurements to ascertain any significant impact on the quality of patient treatment.
4: Results are clear and very likely to be valid.	Five studies: articles reported post-test scores suggesting that medical/nursing students treated their patients more humanistically as a result of the interventions (25, 26, 37, 41, 45).
5: Unequivocal results: reserved for research with clear impact, typically associated with post-test scores and/or successful stories of patient treatment long-term.	No studies: all included articles had a relatively short period of training (often one semester) and the absence of post-test surveys to measure long-term impact.

### Evidence of Measurable Outcomes (Based on Kirkpatrick's Model)

We now consider the outcome levels for each of the articles, noting that some studies addressed more than one outcome at different levels (see Table 4).

**Table 4:** Quality of evidence and evidence of measurable outcomes.

**References**	**Kirkpatrick-based outcome levels**	**BEME strength of evidence scale**
Yang et al. (26)	1, 2b	4
Tsai (40)	1, 2a, 2b	3
Yang and Yang (24)	1	1
Wong et al. (42)	1	3
Wang et al. (45)	1	4
Tseng and Lin (36)	1	2
Lin et al. (43)	1	1
Kan et al. (35)	1, 2a	1
Huang et al. (37)	1, 2a	4
Fan et al. (38)	1, 2a	3
Yang et al. (33)	1, 2a, 2b	3
Liao and Wang (25)	1, 2b	4
Cheng et al. (44)	1	3
Chen and Chou (39)	1	2
Chiou et al. (41)	1, 2a	4
Tsai et al. (46)	1	2
Lin et al. (34)	1,	2

#### Level 1: Reactions and Response

All articles addressed level one outcomes reporting student reactions and responses to medical humanities courses (24–26, 33–46). Participants overwhelmingly reported that the medical humanities courses/programs they experienced might be useful in facilitating their awareness of the humanistic element of medical and nursing professions. Cheng et al. (44), BEME level 3, provides a typical example. Participants enjoyed the training, which related to their needs in the medical education context and organization, and considered it an effective use of their time. In Fan et al. (38), BEME level 3, reported that students recognized the difficulties of learning psychiatry traditionally and how the use of literature can combat these impediments: using the literature to probe human nature and the inner mind of someone with lived experience of mental illness. The intervention not only made psychiatry more accessible but also more appealing.

#### Level 2a: Modification of Attitudes or Perceptions

Six studies [all BEME levels 3 and 4, except 1 study with level 1 of Kan et al. (35)] reported modification of attitudes or perceptions in treating patients in a more humanistic way (33, 37, 40, 41) or creative study (35) and fresher mind in critical thinking (38). Three of these provided community experiences for students with outcomes consistently demonstrating improvement in terms of patient respect and reflective practice. One study provided a narrative medicine program with the outcome not only being a greater improvement in respect and reflective practice but also higher empathy for many participants. However, not all participants developed equally. For example, one study evaluating a narrative medicine program with medical students learning Traditional Chinese Medicine and those learning Western Medicine found that self-development and reflection were more favorable for the Traditional Chinese Medicine student group than for the Western Medicine group (37). Another, focusing on creating a higher awareness of the sense of life, reported that their program contributed to helping medical students gain more mature attitudes toward death and decreased negative emotions toward cadavers (41), it also drew the learning model for medical students in manners dealing with people or clients and matters and attitudes toward difficulties. Finally, one study reported that students gained a greater respect for service, the efforts made by their teachers, the importance of being a volunteer, and the enthusiasm of social interaction through interacting with the elderly community (33).

In terms of improvement in perceptions about study, specifically, in Fan et al.'s study (BEME level 3) in which the post-course outcome comprised students' grades in their fourth-year general psychiatry performance, it was found that students who had attended the course had scores that were significantly higher compared to those who did not. The authors attributed this to a more creative and fresher mind in critical thinking (38).

The second study used an experimental, non-randomly controlled design with a field visit, group writing report, and group assignment as the interventions (35), and was classified as a BEME level 1. At the end of the course, students demonstrated greater creativity in terms of responding to their report-writing remit by using formats such as pictorial storybooks, conversations between a father and a son and movie scripts rather than adopting the more traditional report-writing genre.

It should also be noted that there were some articles (BEME level 1) that sought to achieve level 2a outcomes, including (24, 43, 46) but the results did not show the expected improvements. In particular, two articles (24, 46) used course interventions for medical students, however, their goals of improvement of empathy score and ethical decision-making remained low.

#### Level 2b: Modification of Knowledge and Skills

Four studies were categorized at this level (across BEME levels 3 and 4) in which participants demonstrated modifications in terms of clinical treatment knowledge and skills via relevant humanities activities, such as critical thinking (25, 26), reflective writing, teamwork, cultural awareness, observational skills (26), empathy, or empathic communication (25, 26, 33, 40). The study by Liao and Wang (25), classified at BEME level 4, measured changes in medical students empathy, critical thinking, and reflective writing skills, finding significant differences in aspects of all three domains. In addition to the data on students' reactions to a visual arts program reported under Kirkpatrick Level 1 above, Yang et al. (26) also drew on instructors' notes and evaluations to conclude that students were better able to identify and describe protagonists' emotions following multiple discussions with peers and instructors. The other two studies explored the impact of service-learning and community work on medical students clinical skills: Tsai (40) reported an increased capacity for self-reflection and knowledge of caring for and communicating with vulnerable people, while Yang et al. (33), BEME level 4, noted an increased capacity in students to engage with their communities and work collaboratively on such projects.

#### Level Three: Behavioral Change

There were no articles in our review that aligned with this level.

#### Level Four: Change in Organizational Practice or Patient Outcomes

There were also no articles in our review that aligned with this level.

## Discussion

Our findings suggest that all four rationales outlined in the literature (3) are represented in medical humanities studies relating to Taiwan. However, instrumental and intrinsic rationales dominate over critical or epistemological. This is unsurprising as critical and epistemological rationales are relatively recent perspectives taken up in global medical humanities scholarship (3). This distinction is also reflected in the way the Medical Humanities have been introduced into the Taiwanese curricula, with an emphasis on “first generation” perspectives (13) as either being an antidote to medical science or a way of developing “softer” skills (e.g., communication). Furthermore, this might also be due to the difficulty of Taiwanese educators adopting a critical perspective, questioning the orthodoxy of medicine, the roles of patients vs. caregivers, and separating biology and culture. These ideas can conflict with the traditional values of a Confusist nation. As Taiwan begins to mature with its work in this area, there is considerable scope for expanding research on the medical humanities to include the critical and epistemological perspectives as well.

Similarly, narrative/storytelling, coursework, and fieldwork are the most frequently employed interventions when incorporating the Medical Humanities into Taiwanese medical curricula. Again, we feel that this reflects the nascent nature of the field in Taiwan. As the Medical Humanities gain more traction in the country and become part of the core curricula educators will likely seek out more novel approaches, such as seen in the few studies which explored the use of community-based experiences, exposure to art, and the “silent mentor” program (**see** Table 1). As for exposure to visual art, for example, by using facilitated group discussion of an art image, Shapiro et al. (49) demonstrated that an approach of visual thinking strategies appeared to increase team building as medical interns worked together, challenging each other to form a cohesive idea about the art form studied. Dolev et al. (50) found improved visual diagnostic skills in medical students who participated in art observation workshops through systematic visual training using representational paintings. According to Shapiro et al. (49), students' can develop skills in emotional recognition and cultivate empathy in arts-based conditions. It is suggested that seeing is defined not only as observation of physical signs and features but also as a process of understanding the person and context. Stress reduction for medical professionals through an arts-in-medicine program has also been demonstrated. Indeed, we believe that, by critically examining their rationales for including the Medical Humanities in their curricula, including what is covered and understood by the term Medical Humanities itself, Taiwanese educators will develop their educational repertoire, and consider including culturally sensitive art forms (e.g., traditional, Aboriginal and folk art), bringing them closer to understanding the “other” and human suffering.

### Ways Medical Humanities Outcomes Assessed in Taiwan's Medical Curricula

Our review found that medical humanities outcomes in Taiwan are assessed in a variety of ways, including (and in approximate order of frequency): self-assessments; written methods such as assignments, reflective writing and clinical/field reports; faculty observation/judgment; and content tests or presentations. Other studies used alternative measures, such as academic grades, clinical notes, course surveys, interviews, and a mental health survey. The frequency of self-assessments or reports is reflected in the predominance of outcomes at Level 1 on Kirkpatrick's model (see below for further discussion). On one level, this plurality of methods suggests that Taiwanese medical schools have implemented medical humanities education quite comprehensively. However, it may also suggest a continuing search for appropriate and valid methods of assessing what is recognized as challenging skills and outcomes to assess (17, 51, 52). It should also be noted that these assessment methods were frequently described by the clinical teachers themselves and may not accord with the perspective of those who directly received such assessments: namely, students, clerks, and interns.

### Quality of Evidence and Evidence of Measurable Outcomes

The majority of studies in this review reported findings based on participant reactions to the intervention, that is, Level 1 of Kirkpatrick's model. A small number of studies reported a higher level of outcomes (Levels 2a, 2b), although no study was found which claimed observable changes in the students' themselves in terms of application to daily life after their newly acquired knowwledge/attitude, and at organizational or patient levels. In terms of strength of evidence, there was a spread of studies across Levels 1-4 of the BEME strength of evidence scale, with many using either quantitative or qualitative methods, rather than the approaches necessary for a Level 4 classification, such as mixed methods, diversified participant groups, and more longitudinal and better-aligned assessments such as portfolios. Furthermore, the focus of most of the studies were participants' attitudes, feelings, and knowledge about intended learning outcomes, with few aiming to elicit or evaluate broader changes in participants' real behaviors or impact.

The overall strength of evidence and levels of outcomes of these studies lead us to two broad conclusions: first, students have generally learned the expected skills in accordance with common goals and purposes of the medical humanities as outlined by course designers; second, students generally appreciate the goals and purpose of the course they have taken. While stronger conclusions relating to changes in organizational or patient outcomes are not warranted at this stage, these are nevertheless positive results for the relatively recent adoption of medical humanities in Taiwan. This is also consistent with Ousager and Johannessen (7) findings that most papers on the medical humanities report on participants' reactions and responses to the interventions. Taiwanese studies in the medical humanities are no different in terms of this focus, which likely represents the inherent challenge of assessing interventions whose purported effects are arguably significantly “downstream.” In other words, the desired impacts are hard to measure, hard to attribute to the intervention, and likely influenced by multiple factors (51–53).

### Conceptualizations of the Medical Humanities

Finally, we found a dominance of western definitions of the medical humanities being used in the studies we reviewed. While a useful definition in practical curricular terms, the reliance on western conceptualizations of the medical humanities may have an unintended consequence in overly constraining notions and applications of the medical humanities in non-western contexts. This may be problematic, as it often emerges in practice in a “quasi-Western” form through the use of Western cultural artifacts (via history, philosophy, literature, and art), potentially marginalizing local expressions of cultural diversity not only for patients and society but also for clinical practitioners and students' themselves (13). It is thus important that Taiwan's medical scholars and practitioners be open to refining and articulating their ideas about the medical humanities which may be more clinically and pedagogically appropriate to their culture, society, and values.

### Review Limitations and Strengths

We acknowledge several limitations of our study. Firstly, the lack of clear articulation or attribution of definitions of medical humanities in many studies has meant that details of the curricular intervention were not always clear, so we adopted an inclusive approach. Similarly, the lack of explicit rationales for the use of the medical humanities in many studies meant that we as authors have had to infer what these might be from the details provided of the curriculum, and our inferences may not accord with the (unstated) intentions of the educators. Finally, as we did not search the gray literature (for example, medical school evaluations or conference proceedings) our study may not have identified all potentially relevant studies of the medical humanities outcomes, particularly with the practice of medical humanities in Taiwan.

Despite these limitations, we believe our study offers important insights, such as data on the alignment between the expected outcomes of medical humanities education and the actual outcomes, as reflected in the relevant academic literature. This focus has enabled us to confirm that most evaluations of the medical humanities continue to target student perceptions or knowledge while identifying some studies which do appear to address higher outcome levels and/or provide a stronger basis for claims of impact. At the same time, our focus on a specific national context has enabled us to provide a relatively comprehensive survey of the outcomes and practices related to a relatively homogeneous curriculum, an important factor for a highly contextual educational domain as reflected by medical humanities pedagogy.

## Conclusions

Medical humanities education appears to be growing in importance in Taiwan and the results of this systematic review reflect this development. Nevertheless, a clear and locally produced consensus about the nature and practice of the medical humanities in the Taiwan context remains to be reached. There is also considerable scope to expand the focus of research in the medical humanities from intrinsic and instrumental rationales to critical and epistemological rationales for its adoption in medical education. The main approaches and interventions for delivering the medical humanities in Taiwan include narrative/storytelling, coursework, and fieldwork, along with several other related interventions. The ways medical humanities outcomes are assessed in Taiwan's medical curricula are currently heavily dependent on soliciting the students' perspective. In line with the higher levels of the BEME strength of evidence scale, more diversified participants' backgrounds, mixed methods, and assessments aligned with the outcomes of interest are recommended to produce more compelling evidence of the impact of medical humanities programs. Similarly, studies exploring higher-level outcomes according to Kirkpatrick's model would further advance our understanding of the impact of medical humanities curricula, in particular, the long-term impacts of medical humanities education for the medical students, practitioners to patients, and patient care remain unclear. Longitudinal studies thus should also be encouraged as they should provide clearer evidence of participants' behavioral change. Finally, future studies which broaden the evidence base, such as interviews with clinicians, policymakers, and patients, should shed more light on the implementation and evaluation of humanities education in medical schools.

## Data Availability Statement

The original contributions presented in the study are included in the article/Supplementary Materials, further inquiries can be directed to the corresponding author.

## Author Contributions

BLH, C-DH, and LVM contributed to the development of the study, analysis and interpretation of the data, writing, reviewing and finalizing of the manuscript. K-SC and S-CC participated in the study conceptualization, analyzed the data, and critically revised the manuscript. NC participated in the interpretation of the data and critically revised the manuscript. YSM participated in the analysis and interpretation of the data and critically revised the manuscript. All authors read and approved the final version of the manuscript.

## Funding

This study was supported by the Ministry of Science and Technology, ROC [MOST 107- 2511-H-182-013], [MOST 109-2511-H-182-006] and Chang Gung Memorial Hospital, Taiwan [CDRPG 3J0051].

## Conflict of Interest

The authors declare that the research was conducted in the absence of any commercial or financial relationships that could be construed as a potential conflict of interest.

## Publisher's Note

All claims expressed in this article are solely those of the authors and do not necessarily represent those of their affiliated organizations, or those of the publisher, the editors and the reviewers. Any product that may be evaluated in this article, or claim that may be made by its manufacturer, is not guaranteed or endorsed by the publisher.

## Abbreviations

BEME: Best Evidence Medical Education
Embase: Excerpta Medica database
ERIC: Education Resources Information Center
RQ: Research Question
PRISMA-P: Preferred Reporting Items for Systematic Review and Meta-Analysis Protocols.
