# Supplementary material for: Medical Humanities Education and Its Influence on Students' Outcomes in Taiwan: A Systematic Review
Source: Front Med (Lausanne). 2022 May 16;9:857488. doi: 10.3389/fmed.2022.857488 (PMC9150274; doi:10.3389/fmed.2022.857488)
Supplement: Supplementary file 1 [file Data_Sheet_1.docx]

**Appendix 1**

Search strategy terms from the databases

**Database: PubMed (Best Match Mode)**

Date of Search: 25 February, 2019

1. TOPIC: (“medical trainee”) 14211
2. TOPIC: (“medical train*”) 0
3. TOPIC: (“postgraduate trainee”) 2117
4. TOPIC: (“postgraduate year”) 15840
5. TOPIC: (“undergraduate year”) 15155
6. TOPIC: (“undergraduate trainee”) 1024
7. TOPIC: (“medical student intern*”) 0
8. TOPIC: (“medical student intern 1214
9. TOPIC: (“medical student clerk*”) 0
10. TOPIC: (“medical student clerk) 237
11. TOPIC: (“nursing student”) 46564
12. TOPIC: (“nurse student”) 22068
13. TOPIC: (“nurs* student”) 50259
14. TOPIC: (“health care student”) 64016
15. TOPIC: OR/ 1-14 130963
16. TOPIC: (“medical education”) 457958
17. TOPIC: (“medical educator”) 9339
18. TOPIC (“medical training”) 506949
19. TOPIC (“medical trainer”) 3405
20. TOPIC: (“clinical training”) 352356
21. TOPIC: (“clinical trainer”) 3156
22. TOPIC: OR/ 16-21 711337
23. TOPIC: (“curricula”) 103421
24. TOPIC: (“curriculum”) 1281624
25. TOPIC: OR/ 23-24 102240
26. TOPIC (“medical humanities”) 242471
27. TOPIC (“medical humani*”) 242276
28. TOPIC: OR/ 19-20 242471
29. TOPIC: 15 AND 22 AND 25 AND 28 1361
30. Limit 29 to 2000- 2018 1124
31. Limit 30 to English and Chinese 1077
32. **Limit 31 to Taiwan 14**

**Database: Embase**

Date of Search: 25 February, 2019

1. TOPIC: (“medical trainee”) 116382
2. TOPIC: (“medical train*”) 318203
3. TOPIC: (“postgraduate trainee”) 116382
4. TOPIC: (“postgraduate year”) 14266
5. TOPIC: (“undergraduate year”) 8752
6. TOPIC: (“undergraduate trainee”) 22255
7. TOPIC: (“medical student intern*”) 93095
8. TOPIC: (“medical student intern 3373
9. TOPIC: (“medical student clerk*”) 66710
10. TOPIC: (“medical student clerk) 63
11. TOPIC: (“nursing student”) 23727
12. TOPIC: (“nurse student”) 23727
13. TOPIC: (“nurs* student”) 48883
14. TOPIC: (“health care student”) 99085
15. TOPIC: OR/ 1-14 502874
16. TOPIC: (“medical education”) 318203
17. TOPIC: (“medical educator”) 5235
18. TOPIC (“medical training”) 318203
19. TOPIC (“medical trainer”) 3761
20. TOPIC: (“clinical training”) 288303
21. TOPIC: (“clinical trainer”) 3768
22. TOPIC: OR/ 16-21 10773
23. TOPIC: (“curricula”) 16464
24. TOPIC: (“curriculum”) 83777
25. TOPIC: OR/ 23-24 106898
26. TOPIC (“medical humanities”) 3964
27. TOPIC (“medical humani*”) 32269
28. TOPIC: OR/ 19-20 32269
29. TOPIC: 15 AND 22 AND 25 AND 28 10
30. Limit 29 to 2000- 2018 9
31. Limit 30 to English and Chinese 9
32. **Limit 31 to Taiwan 0**

**Database: ERIC**

Date of Search: 25 February, 2019

1. TOPIC: (“medical trainee”) 349
2. TOPIC: (“medical train*”) 290
3. TOPIC: (“postgraduate trainee”) 107
4. TOPIC: (“postgraduate year”) 918
5. TOPIC: (“undergraduate year”) 13,753
6. TOPIC: (“undergraduate trainee”) 235
7. TOPIC: (“medical student intern*”) 125
8. TOPIC: (“medical student intern”) 126
9. TOPIC: (“medical student clerk*”) 63
10. TOPIC: (“medical student clerk) 63
11. TOPIC: (“nursing student”) 5854
12. TOPIC: (“nurse student”) 3612
13. TOPIC: (“nurs* student”) 8
14. TOPIC: (“health care student”) 6657
15. TOPIC: OR/ 1-14 2588
16. TOPIC: (“medical education”) 24468
17. TOPIC: (“medical educator”) 1528
18. TOPIC (“medical training”) 5986
19. TOPIC (“medical trainer”) 166
20. TOPIC: (“clinical training”) 5431
21. TOPIC: (“clinical trainer”) 205
22. TOPIC: OR/ 16-21 1435
23. TOPIC: (“curricula”) 24834
24. TOPIC: (“curriculum”) 214135
25. TOPIC: OR/ 23-24 223714
26. TOPIC (“medical humanities”) 236
27. TOPIC (“medical humani*”) 0
28. TOPIC: OR/ 19-20 27
29. TOPIC: 15 AND 22 AND 25 AND 28 35881
30. Limit 29 to 2000- 2018 11956
31. Limit 30 to English and Chinese 11956
32. **Limit 31 to Taiwan 104**

**Database: PsycInfo**

Date of Search: 25 February, 2019

1. TOPIC: (“medical trainee”) 56
2. TOPIC: (“medical train*”) 2911
3. TOPIC: (“postgraduate trainee”) 12
4. TOPIC: (“postgraduate year”) 53
5. TOPIC: (“undergraduate year”) 162
6. TOPIC: (“undergraduate trainee”) 10
7. TOPIC: (“medical student intern*”) 112
8. TOPIC: (“medical student intern”) 7
9. TOPIC: (“medical student clerk*”) 2
10. TOPIC: (“medical student clerk) 0
11. TOPIC: (“nursing student”) 107
12. TOPIC: (“nurse student”) 34
13. TOPIC: (“nurs* student”) 203
14. TOPIC: (“health care student”) 364
15. TOPIC: OR/ 1-14 3677
16. TOPIC: (“medical education”) 2936
17. TOPIC: (“medical educator”) 32
18. TOPIC (“medical training”) 2647
19. TOPIC (“medical trainer”) 14
20. TOPIC: (“clinical training”) 6705
21. TOPIC: (“clinical trainer”) 25
22. TOPIC: OR/ 16-21 9965
23. TOPIC: (“curricula”) 341
24. TOPIC: (“curriculum”) 3420
25. TOPIC: OR/ 23-24 3609
26. TOPIC (“medical humanities”) 104
27. TOPIC (“medical humani*”) 214
28. TOPIC: OR/ 19-20 214
29. TOPIC: 15 AND 22 AND 25 AND 28 3
30. Limit 29 to 2000- 2018 2
31. Limit 30 to English and Chinese 2
32. **Limit 31 to Taiwan 0**

**Database: Web of Science**

Date of Search: 25 February, 2019

1. TOPIC: (“medical trainee”) 6333
2. TOPIC: (“medical train*”) 53509
3. TOPIC: (“postgraduate trainee”) 1345
4. TOPIC: (“postgraduate year”) 4414
5. TOPIC: (“undergraduate year”) 10046
6. TOPIC: (“undergraduate trainee”) 485
7. TOPIC: (“medical student intern*”) 6446
8. TOPIC: (“medical student intern”) 748
9. TOPIC: (“medical student clerk*”) 3126
10. TOPIC: (“medical student clerk) 180
11. TOPIC: (“nursing student”) 16386
12. TOPIC: (“nurse student”) 16386
13. TOPIC: (“nurs* student”) 16504
14. TOPIC: (“health care student”) 14871
15. TOPIC: OR/ 1-14 91171
16. TOPIC: (“medical education”) 73740
17. TOPIC: (“medical educator”) 4552
18. TOPIC (“medical training”) 51007
19. TOPIC (“medical trainer”) 1329
20. TOPIC: (“clinical training”) 62908
21. TOPIC: (“clinical trainer”) 1349
22. TOPIC: OR/ 16-21 151207
23. TOPIC: (“curricula”) 41429
24. TOPIC: (“curriculum”) 41429
25. TOPIC: OR/ 23-24 41429
26. TOPIC (“medical humanities”) 915
27. TOPIC (“medical humani*”) 2733
28. TOPIC: OR/ 19-20 2733
29. TOPIC: 15 AND 22 AND 25 AND 28 208
30. Limit 22 to 2000- 2018 189
31. Limit 23 to English and Chinese 176
32. **Limit 24 to Taiwan 2**
