# Supplementary material for: Medical Humanities Education and Its Influence on Students' Outcomes in Taiwan: A Systematic Review
Source: Front Med (Lausanne). 2022 May 16;9:857488. doi: 10.3389/fmed.2022.857488 (PMC9150274; doi:10.3389/fmed.2022.857488)
Supplement: Supplementary file 2 [file Data_Sheet_2.docx]

**Appendix 2**

**Best Evidence Medical Education (BEME) coding scheme for strength of evidence and Kirkpatrick-based outcomes.**

| **BEME strength of evidence scale** | |
| --- | --- |
| 1 | No clear conclusions can be drawn—results based on testimonial evidence of authors |
| 2 | Ambiguous results, there may be a trend—tool(s) for analysis exhibit insufficient power, small sample size, other intervening factors |
| 3 | Conclusions can probably be based on the results—tool(s) used for analysis have sufficient power to assess the outcome |
| 4 | Results are clear and very likely to be true—multiple tools for analysis with sufficient power and explicit triangulation of results |
| 5 | Results are unequivocal |
| **Kirkpatrick-based outcome levels** | |
| Level 1 | PARTICIPATION  Affective reactions and feedback by students (for example: learning experience, course organization, materials, quality of instruction, feelings of pleasure or enjoyment)  *Instruments—surveys, questionnaires, comment forms* |
| Level 2a | MODIFICATION OF ATTITUDES OR PERCEPTIONS  Changes in attitudes towards intervention (for example, sense of personal or professional growth, increase in empathy, new sense of affiliation or connectedness)  *Instruments—observation, pre- and post-tests, interviews, focus groups* |
| Level 2b | MODIFICATION OF KNOWLEDGE AND SKILLS  For knowledge: acquisition of concepts, procedures, or principles. For skills: acquisition of thinking a problem-solving.  *Instruments—observation, pre- and post-tests, interviews, focus groups, course writing samples* |
| Level 3 | BEHAVIORAL CHANGE  Evidence that knowledge and skills learned in course have been applied in subsequent contexts  *Instruments—creative final product (papers, projects, portfolios), surveys, observation* |
| Level 4a | CHANGE IN ORGANIZATIONAL PRACTICE  Attributable changes in organization or delivery of care  *Instruments—alumni surveys, patient and/or employee feedback* |
| Level 4b | BENEFITS TO PATIENTS AND CLIENTS  Improvement in health and well-being of patients as a direct result of classroom intervention  *Instruments—patient feedback* |
